# Characterization of a novel mutant with inhibition of storage root formation in sweet potato

**DOI:** 10.1270/jsbbs.22090

**Published:** 2023-04-27

**Authors:** Hyungjun Park, Tomoko Abe, Hisato Kunitake, Tomonari Hirano

**Affiliations:** 1 Interdisciplinary Graduate School of Agriculture and Engineering, University of Miyazaki, 1-1 Gakuenkibanadainishi, Miyazaki-shi, Miyazaki 889-2192, Japan; 2 RIKEN Nishina Center for Accelerator-Based Science, Wako, Saitama 351-0198, Japan; 3 Faculty of Agriculture, University of Miyazaki, 1-1 Gakuenkibanadainishi, Miyazaki-shi, Miyazaki 889-2192, Japan

**Keywords:** fibrous root, heavy-ion beam, *Ipomoea batatas*, pencil root, storage root

## Abstract

Sweet potato is a widely cultivated crop with storage roots. Although many studies have been conducted on the mechanism of its storage root formation, the details have not been fully elucidated. We screened mutant lines with inhibition of storage root formation to clarify parts of the mechanism. In this study, the process of storage root formation in one of the mutant lines, C20-8-1, was investigated. The inhibition of storage root formation was observed during the early stages of growth. The roots in C20-8-1 did not show histological differences compared to those in wild type. The transition from fibrous roots to pencil roots, which are the developmental stages prior to mature storage root formation, was delayed or inhibited in C20-8-1. The upregulation of starch biosynthesis-related genes and downregulation of lignin biosynthesis genes with storage root swelling were not confirmed in the root of C20-8-1 during the developmental transition stage, suggesting that most of the roots in C20-8-1 are in the pre-transition state toward the storage root swelling. C20-8-1 showed a mutant phenotype during the critical period of storage root swelling initiation, and further clarification of this mutation is expected to provide new insights into storage root formation.

## Introduction

Sweet potato (*Ipomoea batatas* [L.] Lam.) is one of the most important food crops and is cultivated worldwide. Storage root (SR) is an economically important component of sweet potato. SR has been used for table, food processing, alcohol, and industrial purposes because it is rich in carbohydrates, vitamins, and micronutrients (Katayama *et al.* 2017, Panda and Sonkamble 2012). Therefore, the primary target of sweet potato breeding is its SR traits and breeding objectives such as good eating quality, good appearance, resistance to diseases and pests, high yield, and various functional components (Monden and Tahara 2017, Tanaka *et al.* 2017).

Understanding SR formation mechanisms is necessary to consistently obtain high yields and improve breeding efficiency (Yang *et al.* 2023). Numerous comprehensive studies have been conducted from various perspectives, and the anatomical process of SR formation has been well characterized (Tanaka 2016, Wilson and Lowe 1973, Woolfe 1992). SR development begins with stem nodal-derived adventitious fibrous roots (Artschwager 1924, Ma *et al.* 2015, Ravi *et al.* 2014). In the fibrous roots, primary cambiums are formed between protoxylems and protophloems and lignification occurs in the stele (Noh *et al.* 2010, Villordon *et al.* 2009). The development of a pencil root is a turning point in which the expression of the genes in sweet potato fluctuates greatly, lignin content begins to decrease, and starch content begins to increase (He *et al.* 2021, Noh *et al.* 2010). In addition, the circular primary cambium begins to appear around the central metaxylems, and the vascular cambium is differentiated. SR showed anomalous secondary meristems, vascular cambia, and more parenchyma cells with low lignin and high starch contents (He *et al.* 2021, Noh *et al.* 2010).

SR development is a complex process that is regulated by internal and external factors. Several endogenous phytohormones are closely related to the internal factors. The content of indole acetic acid and abscisic acid peaked after the development of fibrous root (Dong *et al.* 2019, Matsuo *et al.* 1988, Nakatani and Komeichi 1991, Noh *et al.* 2010). Cytokinin content increases continuously with the development of SR (Dong *et al.* 2019). Moreover, fibrous roots have significantly lower trans-zeatin riboside content than SR (Matsuo *et al.* 1988, Nakatani and Komeichi 1991). In contrast, nitrogen and potassium nutrients, soil moisture, and air temperature have been identified as external factors (Li *et al.* 2016, Ravi *et al.* 2009). Recently, the molecular mechanisms underlying SR development have been intensively studied, mainly through transcriptome analysis. As mentioned above, the degree of lignification and the extent of starch accumulation in the roots are important for SR initiation and development, and physiological changes have been confirmed at the gene expression level. Lignin biosynthesis-related genes are downregulated during SR bulking (Belehu *et al.* 2004, Firon *et al.* 2013). Starch biosynthesis-related genes are upregulated during SR development with increasing starch content (Dong *et al.* 2019, Firon *et al.* 2013). Furthermore, NAM/ATAF/CUC (NAC) homeobox genes, a transcription factor family, affect SR formation and lignin content (Firon *et al.* 2013), and *IbNAC083* has been identified as a core regulator of SR development in sweet potatoes (He *et al.* 2021). Although these studies have been conducted on sweet potato, the molecular mechanisms underlying the formation and development of SR are less well understood than those in potato tubers (Zierer *et al.* 2021). Thus, more studies are needed to clarify the mechanisms of SR, and mutants of SR formation and development are important resources for understanding these mechanisms. For example, a late-storage root-forming mutant of sweet potato was screened as a somaclonal mutant, and the phenotype in the roots was associated with changes in zeatin riboside content in the root (Nakatani *et al.* 2002). This mutant will help to elucidate the mechanism of hormonal regulation, especially cytokinin contribution, in SR formation.

In a previous study, we screened a mutant line of C20-8-1 from heavy-ion beam-irradiated lines (Park *et al.* 2022). During the mutant screening process, C20-8-1 showed a decrease in DNA content and inhibition of SR formation. To elucidate the details of the inhibitory mechanism of SR, we characterized the total phenotype of C20-8-1 mutant line and analyzed the gene expression involved in the regulation of SR formation. The transition from fibrous roots to pencil roots was delayed or suppressed in C20-8-1, and this early developmental change was supported by changes in gene expression. C20-8-1 is expected to be a new mutant resource for understanding SR mechanisms.

## Materials and Methods

### Plant materials

The heavy-ion beam-irradiated line C20-8-1 was derived from *Ipomoea batatas* ‘Beniharuka’ (Park *et al.* 2022). C20-8-1 line and wild type (WT) of ‘Beniharuka’ were maintained in an in-vitro culture. Acclimatization and cultivation for detailed analysis of the SR formation were performed in a greenhouse under uncontrolled temperature and humidity conditions at the University of Miyazaki, Miyazaki, Japan. The plant materials were transplanted into 10 L of black plastic pots filled with culture soil (n20950392, NAFCO) on June 15, 2021.

Three replicates of each plant were harvested 15, 45, and 90 days after transplanting (DAT). After harvesting, the roots of each plant were cleaned with water. Total weight, total shoot weight, total root weight, and leaf area were measured. The leaf area was measured using ImageJ for the three largest leaves of each plant. The roots were immediately frozen in liquid nitrogen and stored at –80°C or fixed in FAA fixative.

As the yield test for WT and C20-8-1, the acclimatized plants were cultivated in the field at Kushima, Miyazaki, Japan, according to Park *et al.* (2022). The total weight and number of the SRs were measured only for those weighing 50 g or more.

### Histological analysis

Paraffin sectioning was performed on fibrous root samples. The fixed roots were immersed in turns for more than 3 hours; distilled water:ethanol:n-butanol = 50:40:10, 30:50:20, 15:50:35, 0:50:50, 0:30:70, 0:0:100, and 0:0:100. Briefly, the samples were stained with safranin and embedded in paraffin. Paraffin blocks containing the samples were sectioned to a thickness of 15 μm using a microtome. Paraffin sections were extended on glass slides and deparaffinized under the following conditions. The slide glasses were immersed for more than 30 min twice in 100% xylene; xylene:ethanol = 1:1; 100%, 90%, 70%, 50% ethanol; and distilled water. After staining with hematoxylin for 1 min, dehydration was performed in the reverse order of deparaffinization. Thereafter, they were sealed with Entellan new (EX90554761, Merck) sealant and covered with a micro cover glass. Specimens were observed and photographed using an optical microscope (CX41LF, OLYMPUS) and AxioCam208color OSD (ZEISS).

The samples at 45 and 90 DAT were stained with safranin diluent (50% ethanol 5 mL mixed with Safranin solution 10 μL) for 1 min and washed twice with distilled water. Afterward, the sample was stained with fast green diluent (95% ethanol 5 mL mixed with Fast Green FCF (FUJIFILM) 0.01 g) for 10 s and washed twice with distilled water. In the last step, the sample was re-stained with a safranin diluent for 30 s and washed twice with distilled water. Specimens were observed and photographed using a stereomicroscope (SZ2-ILST, OLYMPUS) and a MicroStudio system (Wraymer).

### Quantitative reverse transcription PCR (qRT-PCR) analysis

Total RNA was isolated from all root samples using the RNeasy Plant Mini Kit (Qiagen) and then treated with RNase-free DNase I (Invitrogen). cDNA was reverse-transcribed from 1.5 μg of total RNA in each sample using the SuperScript IV First-Strand Synthesis Kit (Invitrogen) with random hexamers (Invitrogen). The procedures were performed according to the manufacturer’s instructions.

The qRT-PCR reaction consisted of 8.2 μL depc water, 10 μL 2X SYBR qPCR Master Mix (KAPA SYBR FAST qPCR Master Mix (2X) Kit (KAPA Biosystems)), 0.4 μL 10 mM of each primer, and 1 μL of cDNA (20 ng). The PCR procedure was as follows: CFX Connect Real-Time PCR Detection System (Bio-Rad); 95°C for 3 min, 40 cycles of 95°C for 3 s, 60°C for 20 s, and a melting curve was generated ranging from 60°C to 95°C. Three biological replicates and three technical replicates per biological replicate were performed for each experiment. Relative gene expressions were calculated using the 2^–ΔΔCT^ method (Livak and Schmittgen 2001). The primers used in this study are shown in Supplemental Table 1, and *ACT* was used as the reference gene (Tanaka *et al.* 2009).

## Results

### Phenotypic characterization of C20-8-1

At first, we confirmed the yield of the mutant line of C20-8-1 cultivated in the field. The average yield (g) and number of the SRs in WT were 960.0 ± 92.5 S.E. and 4.3 ± 0.5 S.E., respectively, and those in C20-8-1 were 430.0 ± 54.5 S.E. and 2.5 ± 0.3 S.E., respectively. The yield and SR number in C20-8-1 were significantly decreased compared to those in WT (n = 4, *p* < 0.05 by Student’s t-test).

**Fig. 1.**
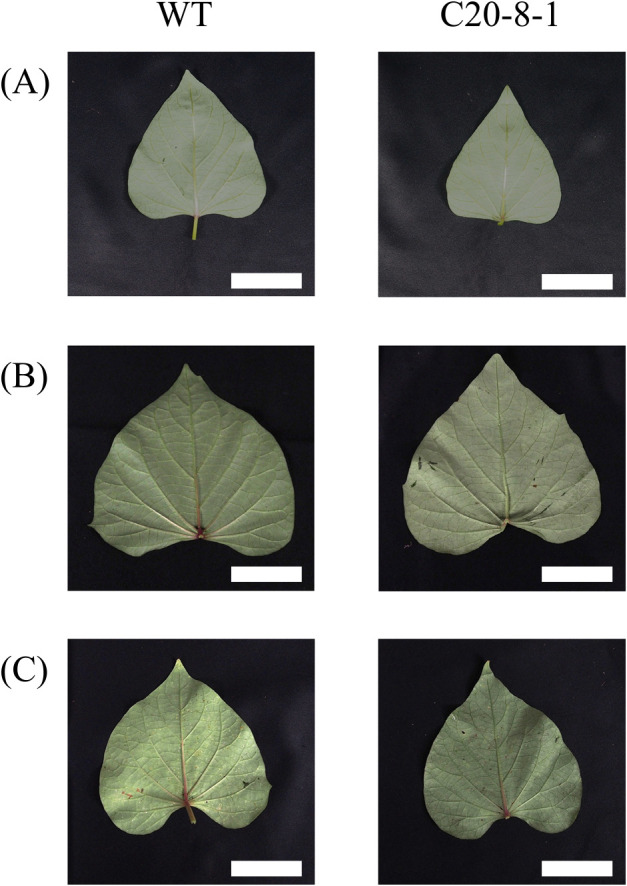
Comparison of leaf phenotype between WT and C20-8-1. (A) 15 DAT, (B) 45 DAT, and (C) 90 DAT. Scale bars = 5cm.

To characterize the detailed phenotype of C20-8-1, the weight of the plant parts of WT and C20-8-1 at early developmental stages was compared (Table 1). There was no difference in the weight of the whole plant throughout the stages. Although the yield and SR number in C20-8-1 decreased compared to those in WT, the total root weight in early developmental stages did not significantly decrease in C20-8-1. Total shoot weight did not also change between WT and C20-8-1, but C20-8-1 had significantly smaller leaves than WT (Fig. 1, Table 1).

### SR formation in C20-8-1

**Fig. 2.**
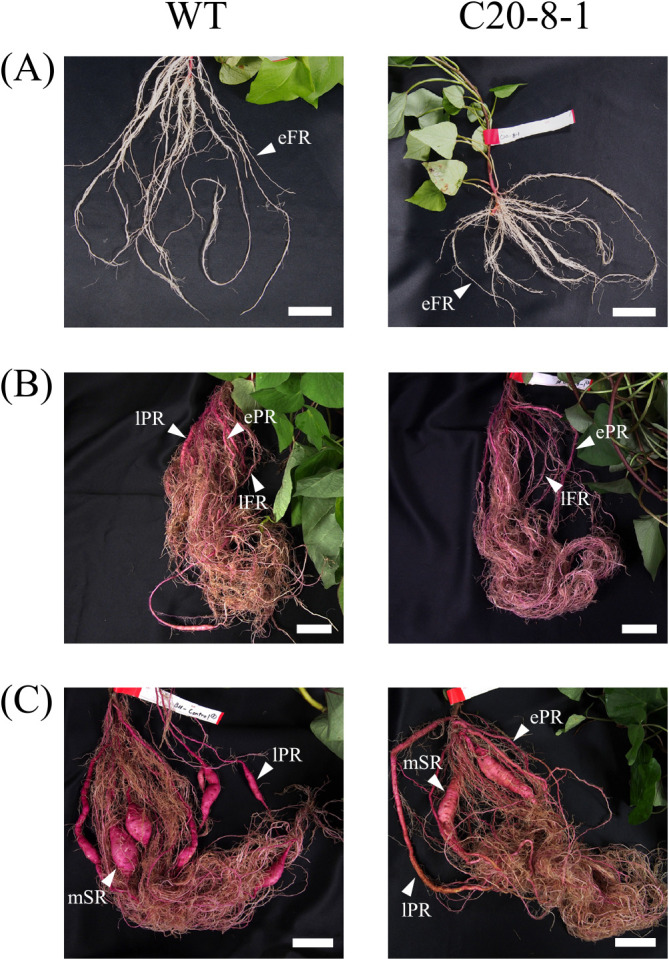
SR formation process of WT and C20-8-1. (A) 15 DAT, (B) 45 DAT, and (C) 90 DAT. eFR, early fibrous root; lFR, late fibrous root; ePR, early-developing pencil root; lPR, late-developing pencil root; mSR, mature storage root. Scale bars = 5cm.

Morphological observations were performed to determine the SR formation process in C20-8-1 (Fig. 2). The morphological classification of sweet potato roots was based on their developmental stages, according to Wang *et al.* (2016). The developmental stages were divided into early fibrous roots (stages 1–3, diameter < 2 mm without anthocyanin accumulation), late fibrous roots (stages 4–8, diameter < 2 mm with anthocyanin accumulation), early-developing pencil roots (stages 9–13, 2 mm ≤ diameter < 5 mm), late-developing pencil roots (stages 14–17, 5 mm ≤ diameter < 20 mm), and mature SRs (diameter ≥ 20 mm).

**Fig. 3.**
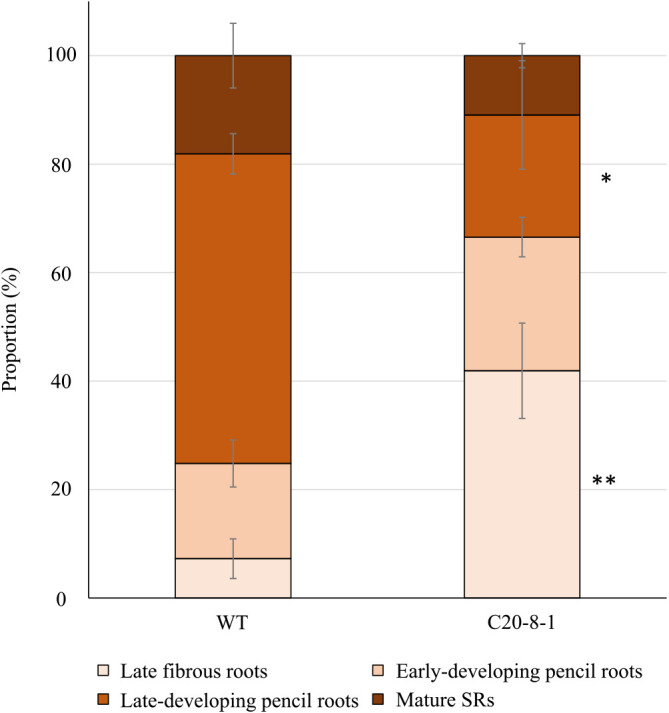
Proportion of roots in the developmental stages at 90 DAT. Each value shows the mean ± S.E. *Value of C20-8-1 is significantly different compared to that of WT in each developmental stage according to t-test (n = 3, *: *p* < 0.05, **: *p* < 0.01).

At 15 DAT, all the roots were categorized as early fibrous roots in WT and C20-8-1 plant (Fig. 2A). At 45 DAT, the roots in WT began to swell, and late-developing pencil roots were observed (Fig. 2B). However, in C20-8-1, all the roots were fibrous roots and several early-developing pencil roots (Fig. 2B). At 90 DAT, both WT and C20-8-1 plants formed late-developing pencil roots and mature SRs (Fig. 2C). When we investigated the proportion of the roots in the late fibrous root stage and later at 90 DAT, the late-developing pencil roots and mature SRs were more than 70% in WT and less than 35% in C20-8-1 (Fig. 3). In particular, WT roots were 57% of the late-developing pencil roots, and C20-8-1 had more than 40% of the late fibrous roots. The total number of the late fibrous roots and later at 90 DAT were 11.7 ± 2.7 S.E. in WT and 12.0 ± 0.5 S.E. in C20-8-1, and there was no significant difference (n = 3, *p* < 0.05 by Student’s t-test).

**Fig. 4.**
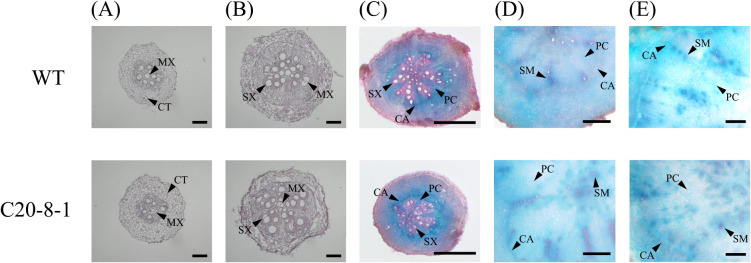
Histological observation of roots in the developmental stages. (A) early fibrous roots, (B) late fibrous roots, (C) early-developing pencil roots, (D) late-developing pencil roots, and (E) mature SRs. CA, cambium; CT, cortex; MX, metaxylem; PC, parenchyma cell; SM, secondary meristem; SX, secondary xylem. Scale bars = (A, B) 200 μm and (C–E) 1 mm.

The inner structure of the roots was observed histologically (Fig. 4). We compared the traverse sections at each developmental stage between WT and C20-8-1 groups. In the fibrous roots of WT and C20-8-1, metaxylems and secondary xylems formed in the steles as prominent components (Fig. 4A, 4B). The early-developing pencil roots of WT and C20-8-1 formed clear circular cambiums (Fig. 4C). In both WT and C20-8-1, secondary meristems and parenchyma cell increases were observed in the steles of the late-developing pencil roots and mature SRs (Fig. 4D, 4E). Safranin staining for lignified cells indicated that occupancy of the lignified cells in the root sections decreased during root swelling from early-developing pencil roots to mature storage roots (Fig. 4C–4E). From these results, there were no obvious structural differences between WT and C20-8-1.

### Comparison of relative expression levels of the genes related to SR formation

**Fig. 5.**
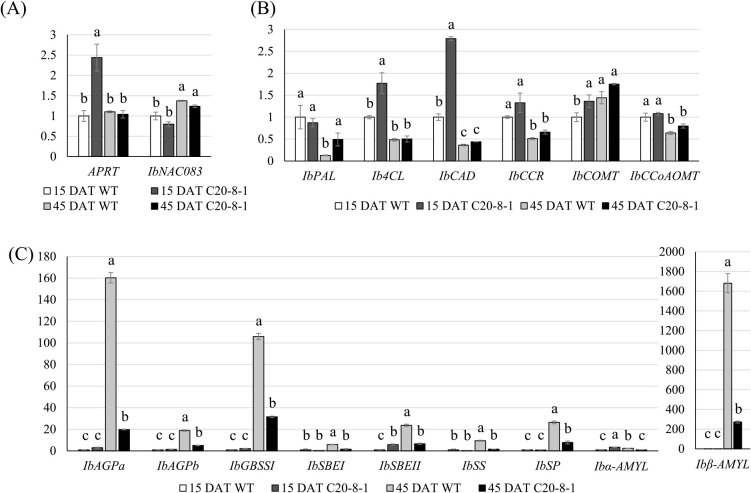
Changes in the transcriptional levels of SR-formation related genes at 15 DAT and 45 DAT. The genes are categorized as (A) cytokinin-related gene and transcription factor, (B) lignin biosynthesis-related genes, and (C) starch metabolism-related genes. Each value is expressed as the mean ± S.E. relative to the respective 15 DAT WT value set at 1.00. Different letters represent significant differences according to Tukey’s test (n = 3, *p* < 0.05).

We focused on the early development of SR in C20-8-1, and qRT-PCR was used to analyze the SR development-related gene expression levels at 15 and 45 DAT. The root samples for RNA extraction at 15 DAT consisted of early fibrous roots from WT and C20-8-1 plants. Those at 45 DAT consisted of late fibrous to late-developing pencil roots in WT and late fibrous to early-developing pencil roots in C20-8-1. We selected the genes based on previous reports and a primer test in WT of ‘Beniharuka’ (Supplemental Table 1). In the hormone-related gene and transcription factor, expression of the *APRT* gene, which is related to cytokinin activation, in C20-8-1 was 2.4-fold higher than that in the WT, with a significant difference at 15 DAT (Fig. 5A). The expression of *IbNAC083*, which is a key regulator of SR swelling, increased from 15 DAT to 45 DAT in WT and C20-8-1, and there was no significant difference in the expression levels at each stage (Fig. 5A). Most of the lignin biosynthesis-related genes decreased from 15 DAT to 45 DAT in WT (Fig. 5B). Among the lignin biosynthesis-related genes, some genes showed higher expression levels in C20-8-1 than in WT: *Ib4CL*, *IbCAD*, and *IbCOMT* genes at 15 DAT and *IbPAL* at 45 DAT (Fig. 5B). In the starch metabolism-related genes at 15 DAT, *IbSBEII* and *Ibα*-*AMYL* showed a higher expression in C20-8-1 than in WT (Fig. 5C). Although the expression of all the genes related to starch metabolism in WT showed remarkable increase from 15 DAT to 45 DAT, the upregulation is suppressed in C20-8-1 at 45 DAT (Fig. 5C).

## Discussion

We applied heavy-ion mutagenesis to ‘Beniharuka’ and obtained mutant candidates with phenotypes of high yield and inhibition of SR formation (Park *et al.* 2022). One of the candidates, C20-8-1, was screened as a line with decreased DNA content and inhibition of SR formation. In this study, we focused on the early developmental stages of SR formation in C20-8-1 to reveal details of the inhibition mechanisms. Based on morphological and histological analysis of the roots, delay in SR development was observed from 45 DAT, and the late-developing pencil roots and mature SRs significantly decreased at 90 DAT in C20-8-1 (Figs. 2, 3). The total numbers of the late fibrous roots and later at 90 DAT were not different between WT and C20-8-1, suggesting that the induction of late fibrous roots was not inhibited in C20-8-1. Moreover, there was no obvious difference in the inner structure of the roots during SR formation (Fig. 4). Therefore, the key process for the inhibition of SR formation in C20-8-1 can be interpreted as a transition from fibrous roots to pencil roots.

For molecular characterization of the change in the SR formation process, the expression levels of the genes related to SR formation and development were compared between WT and C20-8-1, and variation in expression was observed in several genes (Fig. 5). *APRT*, one of the differentially expressed genes, showed a significantly high expression level at 15 DAT (Fig. 5A). *APRT* is involved in cytokinin conversion from active free bases to inactive nucleotides (Zhang *et al.* 2013). A late-storage root-forming mutant of sweet potato showed a decrease in zeatin riboside content (Nakatani *et al.* 2002). Therefore, cytokinin activity may be low in the fibrous roots of C20-8-1 at 15 DAT and may be related to SR formation. The expression of *IbNAC083* in C20-8-1 showed a decreasing trend compared to WT, but not significant. *IbNAC083* has been identified as a key regulator of bulking in sweet potatoes, and downregulation of *IbNAC083* by RNAi inhibits SR formation (He *et al.* 2021). Although the RNAi transgenic plants and C20-8-1 plants showed a similar phenotype, the inhibition of SR formation in C20-8-1 would not be due to changes in the pathway involving *IbNAC083*. He *et al.* (2021) reported that the critical period of SR swelling initiation was identified as the early-developing pencil root period (Stage 10). Lignin content begins to decrease in the SR swelling period (He *et al.* 2021, Noh *et al.* 2010), and upregulation of lignin biosynthesis genes leads to reduced SR production (Wang *et al.* 2016). The developmental stage is also a transition period to initiate rapid starch accumulation and upregulate starch biosynthesis-related genes in the SR (Dong *et al.* 2019, Firon *et al.* 2013, He *et al.* 2021, Li and Zhang 2003, Singh *et al.* 2021). The expression changes on the lignin biosynthesis and starch biosynthesis-related genes from 15 DAT to 45 DAT in WT were consistent with the previously reported changes associated with the SR swelling (Fig. 5B, 5C). However, four genes involved in the general phenylpropanoid pathway and lignin biosynthesis pathway (Dong and Lin 2021), *Ib4CL*, *IbCAD*, and *IbCOMT* genes at 15 DAT and *IbPAL* at 45 DAT, showed the higher expression in C20-8-1 than in WT (Fig. 5B), suggesting that lignin synthetic activity is high in the fibrous roots of C20-8-1, especially at 15 DAT. Moreover, all starch biosynthesis-related genes at 45 DAT in C20-8-1 showed low levels of expression compared with those in WT (Fig. 5C). The starch metabolism activation would be delayed in C20-8-1. The expression variations on the lignin biosynthesis and starch biosynthesis-related genes in C20-8-1 are interpreted that most of the roots in C20-8-1 are in the pre-transition state toward the SR swelling and support the idea that inhibition or delay of the developmental transition from fibrous roots to pencil roots leads to low yields in C20-8-1.

In addition to the SR phenotype, there was a reduction in leaf area in C20-8-1 (Fig. 1, Table 1). Since the total plant weight and shoot weight in C20-8-1 did not change from those of WT, remarkable growth inhibition was not observed. Leaf size in sweet potato negatively correlated with single-leaf net photosynthesis, and single-leaf net photosynthesis did not correlate with the yield of sweet potato (Bhagsari 1990). Therefore, it is difficult to conclude that the significantly smaller leaf area in this study directly affected the development of the inhibited tuberous root. For sweet potato yield, the translocation rates of photosynthetic products are more important than the photosynthetic rates (Bouwkamp and Hassam 1988, Hahn 1977, Wilson 1977). Therefore, an evaluation of the source-sink relationship in C20-8-1 is needed.

In this study, we characterized a novel mutant with a delay or suppression of SR swelling initiation. Gene deletions associated with the decrease in DNA content in C20-8-1 may be thought to inhibit SR formation. Knockout of the key genes mentioned above was not observed in C20-8-1. Further clarification of the details of the mutation in the genome and identification of the gene responsible for the phenotype is expected to provide new insights on SR formation.

## Author Contribution Statement

Conceptualization, T.H. and H.K.; methodology, T.H., H.P., T.A., and H.K.; investigation, H.P., T.H., H.K., and T.A.; writing-original draft preparation, H.P. and T.H.; writing-review and editing, T.A. and H.K.

## Supplementary Material

Supplemental Table

## Figures and Tables

**Table 1.** Phenotypic data according to the DAT

	line	15 DAT	45 DAT	90 DAT
Total weight (g)	WT	39.3 ± 4.6	340.0 ± 15.3	496.7 ± 73.1
	C20-8-1	34.4 ± 1.8^n.s.^	343.3 ± 12.0^n.s.^	486.7 ± 29.1^n.s.^
Shoot weight (g)	WT	33.3 ± 3.9	266.7 ± 17.6	316.7 ± 63.3
	C20-8-1	29.0 ± 1.2^n.s.^	296.7 ± 14.5^n.s.^	336.7 ± 57.0^n.s.^
Root weight (g)	WT	5.5 ± 0.7	73.3 ± 14.5	180.0 ± 10.0
	C20-8-1	4.4 ± 0.4^n.s.^	46.7 ± 3.3^n.s.^	150.0 ± 30.6^n.s.^
Leaf area (cm^2^)	WT	64.8 ± 2.1	121.0 ± 8.1	102.9 ± 7.3
	C20-8-1	47.6 ± 0.7**	99.3 ± 1.5*	80.0 ± 3.2*

Each value is expressed as the mean ± S.E.. * Values are significantly different compared to that of WT using the t-test (n = 3 for the weights and n = 9 for the leaf area, *: *p* < 0.05, **: *p* < 0.01). n.s., not significant.
