# Supplementary material for: Characterization of a novel mutant with inhibition of storage root formation in sweet potato
Source: Breed Sci. 2023 Apr 27;73(2):212–8. doi: 10.1270/jsbbs.22090 (PMC10316310; doi:10.1270/jsbbs.22090)
Supplement: Supplementary file 1 — Supplemental Table [file 73_212_s1.pdf]

Supplemental Table 1 Primers used in this study

Gene	Forward primer (5'-3')	Reverse primer (5'-3')	Reference
Actin (*ACT*)	TGTTAGCAACTGGGATGATATGG	GGATAGCACAGCCTGAATAGC	Tanaka *et al.* 2009
Adenine phosphoribosyl transferase (*APRT*)	CGTGAAATGTGGCAGTGGG	AGCCAGGATTCAATTTTCTGCT	Dong *et al.* 2019
Starch phosphorylase (*IbSP*)	GTGTGACACCAAGAAGATGGA	CGCAGTTCTGCCAACTTTTC	Singh *et al.* 2021
NAM/ATAF/CUC 083 (*IbNAC083*)	CGGATGAGGAGCTTGTGGTT	AGCTCTGCTTGACCTGTTCC	He *et al.* 2021
Phenylalanine ammonia-lyase (*IbPAL*)	GGCGAGCACGAGAAGAATGT	ATGGCAGGGTTTCCGTTCTC	He *et al.* 2021
4-coumarate-CoA ligase (*Ib4CL*)	TATTTTCCGATCGAGGTTGC	ACTTTCCGGCAAATCAAATG	He *et al.* 2021
Cinnamyl alcohol dehydrogenase (*IbCAD*)	AGCTGGTAATGGTTGGCATC	TCCAAAGCCGTGTTGACATA	He *et al.* 2021
Cinnamoyl-CoA reductase (*IbCCR*)	GCAGAGATAACGGCCAGAAG	TTGCTACAACCCACCATCAA	He *et al.* 2021
Caffeic acid/5-hydroxyferulic acid O-methyltransferase (*IbCOMT*)	AAACGGGAAAGTGATCGTTG	CCATGATCCAAGTGTTGACG	He *et al.* 2021
Caffeoyl-CoA O-methyltransferase (*IbCCo-AOMT*)	CCGGTTCTTGACCAGATGAT	TTCCACAGGGTGTTGTCGTA	He *et al.* 2021
ADP glucose pyrophosphorylase a (*IbAGPa*)	TCGACGGTGATGTTAGCAAG	AACAGCCTTTGGAGAAACGA	He *et al.* 2021
ADP glucose pyrophosphorylase b (*IbAGPb*)	GACAAGAACGTAAGGATTGGGA	CGAATGGTTGCTTTCTCCAT	He *et al.* 2021
Granule bound starch synthase I (*IbGBSSI*)	CAGTTGGTTTGCCAGTTGAC	ACGTTGAACTTTGCCACTCC	He *et al.* 2021
Starch branching enzyme I (*IbSBEI*)	GGTTTACGGGTCTTGATGGA	AACAGCCTGCTATCCCACAC	He *et al.* 2021
Starch branching enzyme II (*IbSBEII*)	CTTCCCTGAAGCCATAACCA	CCATTTGCCAATCCTCATCT	He *et al.* 2021
Starch synthase (*IbSS*)	CGGTTCACTTTGCTTTGTCA	CATTGTGTGGGCGATACTTG	He *et al.* 2021
α-amylase (*Ibα-amyl*)	CTGCATTTTTGTTCCTGCAA	TTCGATGCGTCCAAGTCATA	He *et al.* 2021
β-amylase (*Ibβ-amyl*)	AGACTGGAAGGAGGCTGTGA	TGTTGGCTTCTTCGAGGACT	He *et al.* 2021
